# Properties of CO_2_ Micro-Nanobubbles and Their Significant Applications in Sustainable Development

**DOI:** 10.3390/nano15161270

**Published:** 2025-08-17

**Authors:** Zeyun Zheng, Xingya Wang, Tao Tang, Jun Hu, Xingfei Zhou, Lijuan Zhang

**Affiliations:** 1 School of Physics Science and Technology, Ningbo University, Ningbo 315211, China; zhengzeyun2024@sari.ac.cn; 2 Shanghai Synchrotron Radiation Facility, Shanghai Advanced Research Institute, Chinese Academy of Sciences, Shanghai 201204, China; wangxingya@sari.ac.cn; 3 Low-Carbon Conversion Science and Engineering Center, Shanghai Advanced Research Institute, Chinese Academy of Sciences, Shanghai 201210, China; tangt@sari.ac.cn; 4 University of Chinese Academy of Sciences, Beijing 100049, China; 5 Xiangfu Laboratory, Jiaxing 314102, China; hujun64@shu.edu.cn; 6 Institute of Materiobiology, College of Science, Shanghai University, Shanghai 200444, China

**Keywords:** carbon dioxide, micro-nanobubbles, nanobubble technology, eco-friendly nanomaterials

## Abstract

As an important part of global carbon neutrality strategies, carbon dioxide (CO_2_) capture, utilization, and storage technologies have emerged as critical solutions for reducing carbon emissions. However, conventional CO_2_ applications, including food preservation, industrial synthesis, and enhanced oil recovery, face inherent limitations such as suboptimal gas–liquid mass transfer efficiency and inadequate long-term stability. Recent advancements in CO_2_ micro-nanobubbles (CO_2_ MNBs) have demonstrated remarkable potential across multidisciplinary domains, owing to their distinctive physicochemical characteristics encompassing elevated internal pressure, augmented specific surface area, exceptional stability, etc. In this review, we try to comprehensively explore the unique physicochemical properties of CO_2_ MNBs and their emerging applications, including industrial, agricultural, environmental, and energy fields. Furthermore, we provide a prospective analysis of how these minuscule bubbles can emerge as pivotal in future technological innovations. We also offer novel insights and directions for research and applications across related fields. Finally, we engage in predicting their future development trends as a promising technological pathway for advancing carbon neutrality objectives.

## 1. Introduction

With the rapid advancement of global industrialization and urbanization, anthropogenic impacts on the natural environment have escalated significantly. The massive combustion of fossil fuels and exploitation of non-renewable resources have established carbon dioxide (CO_2_) as the dominant greenhouse gas emission, driving critical issues, including global climate warming, increased frequency of extreme weather events, ecosystem degradation, and biodiversity loss [1,2]. To address these pressing concerns, the international community has advocated for low-carbon initiatives to mitigate greenhouse gas emissions, optimize energy efficiency, develop renewable energy systems, and facilitate green economic transitions. Under this background, atmospheric CO_2_ reduction has become a focal point of environmental governance. Existing technologies such as carbon capture and storage (CCS), carbon capture, utilization, and storage (CCUS), CO_2_-enhanced oil recovery (CO_2_-EOR), and CO_2_ geological sequestration (CGS) face significant limitations [3]. These approaches not only fail to align with low-carbon principles but may exacerbate environmental burdens through groundwater contamination risks from CO_2_ leakage, persistent chemical residues in reservoirs, and induced micro-seismic activity. Furthermore, their implementation incurs substantial energy penalties and economic costs. For instance, compressing 80% of CO_2_ emissions in coal-fired power plants increases energy consumption by 24–40% [4]. In China, CCS adoption escalates electricity generation costs by 30–50% due to extra power and steam consumption, with post-combustion capture costs reaching USD 24.3 per ton at the Huaneng Beijing plant, achieving merely 80–85% capture efficiency [5]. These energy-intensive, cost-prohibitive, and low-return characteristics hinder industrial adoption and national-scale implementation.

Recent advancements in micro-nanobubbles (MNBs) have revealed unique physicochemical properties distinct from conventional macro-bubbles and may provide significant contributions in decreasing carbon pollution and efficient utilization of CO_2_. MNBs are generally defined as gas bubbles with diameters ranging from micrometers (μm) down to nanometers (nm). MNBs are classified into two primary types: surface MNBs attached to solid interfaces and bulk MNBs suspended within liquid media [6,7,8]. This study focuses specifically on bulk MNBs. A key characteristic of MNBs is their exceptional stability: contrary to the rapid rupture predicted for bubbles within microseconds by the classical Epstein–Plesset equation, numerous experiments demonstrate that MNBs can maintain their structure in aqueous solutions for hours or even days without readily coalescing or rupturing [9,10,11]. This long-lasting stability, far exceeding theoretical expectations, represents a core advantage. MNBs also typically sustain high internal pressure, forming the basis for their distinct physical and chemical behaviors [12]. Furthermore, they possess a significantly increased specific surface area, which greatly enhances their contact efficiency with surrounding substances such as pollutants or reactants [13]. Given the direct relationship between gas mass transfer efficiency and gas–liquid interfacial contact area, this high specific surface area significantly boosts the gas–liquid mass transfer efficiency of MNBs, enabling them to efficiently promote gas dissolution and transfer while prolonging gas residence time in liquids [14]. For instance, experiments by Matsumoto confirmed that O_3_ MNBs improve ozone mass transfer efficiency and extend its half-life [15]. Additionally, OH^−^ ions are typically adsorbed onto the gas–liquid interface of MNBs, resulting in a negative surface charge[16]. At near-neutral pH conditions, the Zeta potential of Air MNBs, O_2_ MNBs, and N_2_ MNBs typically ranges from approximately −27 to −45 mV [17]. These properties have prompted MNB applications in diverse fields, including biomedicine, wastewater treatment, crop enhancement in agriculture, surface cleaning, mineral flotation, food production, etc. [18,19,20,21,22,23,24]. Notably, the advantages of using MNBs include simple material preparation, low cost, and environmental friendliness. This may provide solutions to environmental pollution challenges brought about by various fields, and is in line with the current low-carbon and environmental protection concept. For instance, in treating black and odorous river water, Hong et al. found that under identical aeration intensity, MNB aeration produced significantly higher dissolved oxygen levels (reaching 9.87 mg/L at 60 min) compared to ordinary aeration (6.54 mg/L). Furthermore, the maximum removal rates for COD, NH_3_-N, geosmin, and 2-methylisoborneol were 12%, 10%, 16%, and 12% higher, respectively [25]. Similarly, when treating simulated wastewater containing azo dyes, Chu et al. observed that O_3_ MNBs increased the total mass transfer coefficient and TOC removal rate by 80% and 30%, respectively [26]. Liu et al.’s experiments on printing and dyeing wastewater treatment revealed that MNB air flotation technology not only enhanced pretreatment efficiency and reduced flocculant dosage but also increased the removal rates of oil, COD, and chromaticity by 40%, 30%, and 110%, respectively, compared to traditional air flotation processes [27].

**Figure 1:**
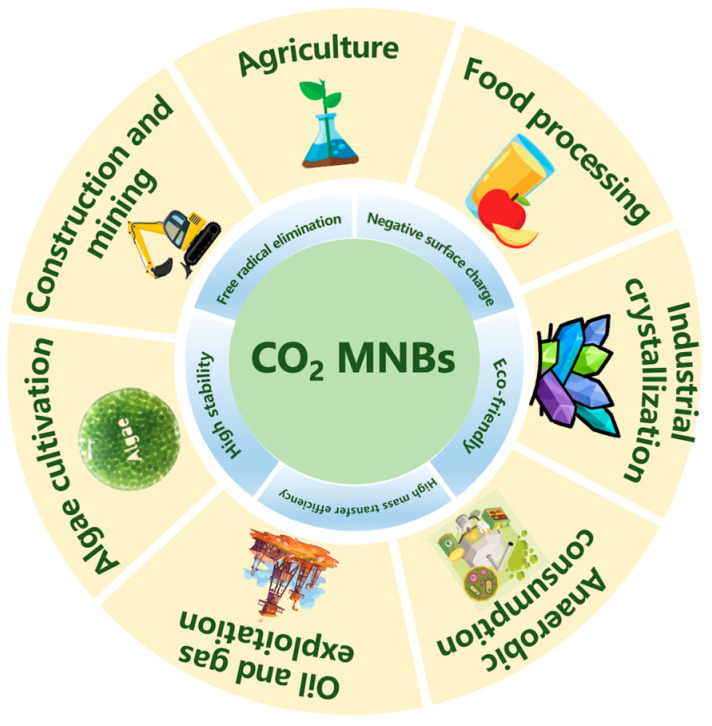
Properties of CO_2_ MNBs and their significant applications.

Herein, we systematically review the unique physicochemical properties of CO_2_ MNBs and their potential applications in various fields. Figure 1 presents all the content involved in this review. The characteristics of CO_2_ MNBs were first deeply analyzed in terms of stability, mass transfer efficiency, surface charge, and elimination of hydroxyl radical. Then, the specific applications in seven major fields were mainly discussed: agricultural planting, food processing, oil and gas extraction, construction mining, anaerobic digestion, microalgae cultivation, and industrial crystallization. It demonstrates CO_2_ MNB technology’s triple advantages: process efficiency amplification, energy demand reduction, and operational cost minimization. These studies underline CO_2_ MNBs’ transformative potential in green manufacturing, environmental engineering, and biotechnological applications, positioning them as key enablers for achieving carbon neutrality and circular economy objectives.

## 2. Properties of CO_2_ MNBs

### 2.1. High Stability

MNBs are distinguished from macroscopic bubbles due to their unique physicochemical properties, and their submicron size confers extraordinary stability to the system, with their survival time in aqueous media reported in the literature to be up to tens of days or even weeks [28,29]. However, significant differences lie in the stability performance of CO_2_ MNBs. Table 1 summarizes the lifespan, pH, zeta potential, and key findings of CO2MNBs generated using different methods. When prepared via the nanobubble film method, CO_2_ MNBs with ~60% population collapse within 72 h post-formation and near-complete dissolution by 5 days [30]. Studies using the periodic pressure change method revealed that CO_2_ MNBs exhibited markedly reduced stability compared to N_2_ and O_2_ MNBs, with a lifetime of less than 48 h [9]. Contrasting stability profiles emerged in hydrodynamic cavitation-synthesized MNBs (N_2_, O_2_, Ar + 8% H_2_, air, and CO_2_) across aqueous matrices. In deionized water, CO_2_ MNBs persisted for 5 days, whereas N_2_/O_2_-based MNBs maintained stability for 60 days. Remarkably, this trend reversed in ionic environments (NaCl, CaCl_2_, and AlCl_3_): CO_2_ MNBs achieved 14-day stability, while other MNBs underwent complete dissolution within 2 weeks [31]. Although different preparation methods may lead to differences in the lifetimes of CO_2_ MNBs in deionized water, all studies consistently showed that the lifetimes of CO_2_ MNBs in deionized water were significantly shorter than those of other MNBs. Notably, researchers attribute the shorter stability of CO_2_ MNBs relative to other gas species to their chemical reactivity with water, as illustrated by the following equations [30,31]:

$${CO}_{2}(aq)+H_{2}O⇌H_{2}{CO}_{3}\;\;\;\frac{H_{2}{CO}_{3}}{{CO}_{2}(aq)}=1.7\times 10^{-3}\;at\;298K$$

$$H_{2}{CO}_{3}⇌H^{+}+{{HCO}_{3}}^{-}\;\;\;K_{a1}=\frac{\left[H^{+}\right]\left[{HCO}_{3}^{-}\right]}{\left[{CO}_{2}\right]+\left[H_{2}{CO}_{3}\right]},pK_{a1}=6.35$$

$${{HCO}_{3}}^{-}⇌H^{+}+{{CO}_{3}}^{2-}\;\;\;K_{a2}=\frac{\left[H^{+}\right]\left[{CO}_{3}^{2-}\right]}{\left[{HCO}_{3}^{-}\right]},pK_{a2}=10.33$$

This dissolution process generates carbonic acid, thereby acidifying the solution. Cerrón-Calle documented that CO_2_ MNB generation in an initial pH 5.8 solution caused rapid acidification, with pH dropping to 4.5 within 5 min and stabilizing at 4.0 [30]. These observations align with findings from others, all confirming that the CO_2_ MNBs system has a significant acidification effect [22,31,32,33,34].

**Figure 2:**
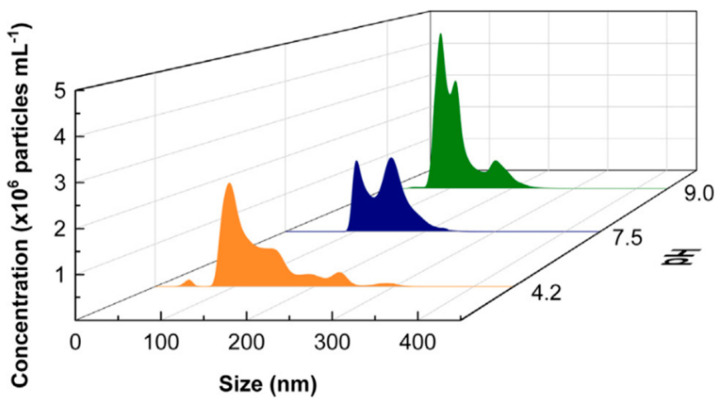
NTA shows the size distribution and concentration of CO_2_ MNBs collected 50 min after generation in solutions with different pH values (4.2, 7.5, and 9.0) [30].

Cerrón-Calle further investigated the pH effect on CO_2_ MNBs size by titrating NaOH into a pH 4.2 CO_2_ MNB solution to systematically raise pH to 7.5 and 9.0 [30]. This manipulation induced a distinct MNBs’ size (Figure 2):

- At pH 4.2: Dominant size distribution 150–250 nm;
- At pH 7.5: Decrease in quantity, and the size distribution is concentrated between 100 and 150 nm;
- At pH 9.0: An increase in quantity, and the size distribution is concentrated between 70 and 100 nm.

The researchers interpreted this phenomenon as pH-driven gas transfer from bubbles to solution, ultimately leading to bubble dissolution. This phenomenon was amplified in low-CO_2_-concentration systems, where MNBs’ shrinkage not only reduced diameters but also supplied dissolved carbon for reactions.

Supporting this mechanism, Takemura demonstrated in shallow aquifers that CO_2_ MNB stability is governed by CO_2_ solubility rather than electrostatic repulsion [35]. This discovery reveals the essence of the anomalous stability of CO_2_ MNBs in salt solutions at the molecular scale—the increase in ionic strength affects the gas–liquid mass transfer kinetics by altering the CO_2_ dissolution equilibrium (CO_2_ (aq) ⇌ CO_2_ (g)), rather than through the compression of the double electric layer effect. The stability of CO_2_ MNBs prepared in the salt solution environment was significantly improved, which also confirmed this conclusion [31].

Therefore, their real lifetime is easily influenced by some factors such as production methods, different pH and ionic compositions, etc. Practical applications may require a continuous CO_2_ MNB generator to sustain their concentration.

### 2.2. High Mass Transfer Efficiency

The gas mass transfer rate depends on the interfacial contact area between the gaseous and liquid phases [16]. Notably, the gas–liquid interfacial area provided by CO_2_ MNBs has been demonstrated to exceed conventional macro-bubbles by several orders of magnitude. This enhanced interfacial characteristic directly translates to superior mass transfer performance. Song et al. observed that CO_2_ MNBs water exhibited a longer nuclear magnetic resonance relaxation time than deionized water, indicating enhanced CO_2_ MNBs water mobility [36]. When evaluating CO_2_ MNBs’ systems through the mass transfer coefficient, experimental measurements revealed an 11-fold enhancement compared to traditional bubbling systems under equivalent gas conditions [30]. Moreover, as the bubble size decreases, the CO_2_ MNBs continuously contract under the action of surface tension. The internal pressure of the bubbles is relatively high, and the mass transfer rate is higher.

### 2.3. Negative Surface Charge

CO_2_ MNBs exhibit surface negative charges, aligning with reported interfacial properties of other gaseous MNBs [17,30,37,38]. The elevated zeta potential magnitude (|ζ|) enhances MNBs’ stability through intensified electrostatic repulsion between adjacent MNBs. Notably, while all reported ζ-values for CO_2_ MNBs fall within the negative range (−2.68 mV to −27 mV), a distinct dichotomy emerges: some experimental data show that their zeta potential values are in the strongly negative charge range from −8 to −27 mV, while the negative charge range recorded by other studies is relatively weak (−2.68 to −5.91 mV) [30,32,34,36,39]. This magnitude difference may be closely related to the characteristics of the solution environment (such as ionic strength, pH value) and the specificity of the bubble generation method.

Time-dependent analyses reveal dynamic interfacial charge evolution. During generation (10–70 min), ζ-potential progressively shifts from −2.68 mV to −3.05 mV concomitant with pH reduction (4.43→4.23), suggesting acid-enhanced charge density and nucleation efficiency [30]. Similarly, during storage, |ζ| amplification peaks at −19 mV after 7 days, correlating with pH elevation via CO_2_ outgassing at the gas–liquid interface [39]. The pH-mediated charge modulation mechanism exhibits universality. Abdella et al. confirmed air/O_2_/N_2_ MNBs dependence for pH: lower pH reduces |ζ|, whereas alkaline conditions intensify surface electronegativity and stability [17]. This consistency underscores the generalized nature of pH-responsive interfacial charge regulation in MNB systems.

### 2.4. Inhibition and Synergistic Effect of CO_2_ MNBs on Hydroxyl Radical Generation

The longstanding controversy regarding whether MNBs can generate hydroxyl radicals remains unresolved: while some experiments have detected hydroxyl radicals in water containing high-concentration MNBs [40,41], others have failed to observe detectable signals [42,43]. For these contradictory findings, Liu et al. postulated that the observed hydroxyl radical signals might originate from H_2_O_2_ produced through hydrodynamic cavitation during MNB generation, rather than the direct free radical itself [44].

CO_2_ MNBs generate radicals through Brownian motion-induced collapse, attributed to their low absolute zeta potential [33]. Although limited radical production was observed in acidic ethanol solutions (pH = 5), electron spin resonance analysis of CO_2_ mixed with hydrogen-containing gases (8% H_2_/Ar) revealed no detectable peaks for either superoxide anion radicals or hydroxyl radicals. This observation aligns remarkably with this study: sequential introduction of H_2_ and CO_2_ MNBs synergistically maximized radical elimination, whereas reverse sequencing (CO_2_ followed by H_2_) paradoxically increased both radical and superoxide anion concentrations. This bidirectional regulatory effect was similarly observed in superoxide anion inhibition patterns [45]. Ultrasonic collapse experiments further highlight the unique behavior of CO_2_ MNBs: unlike air, O_2_, or N_2_ MNBs that substantially generate reactive oxygen species, CO_2_ MNBs exhibit negligible hydroxyl radical production under comparable conditions [22].

Collectively, these findings demonstrate that CO_2_ MNBs possess dual functionality–not only suppressing radical generation during collapse, but also actively scavenging free radicals through synergistic interactions with H_2_ MNBs. This dual-action mechanism provides critical support for their enhanced biocompatibility and environmental remediation potential.

**Table 1:** Preparation methods and properties of CO_2_ MNB.

Preparation Method	Solvent	Characterization Method	Lifetime	pH Value	Average Bubble Size	Zeta Potential	Dissolved CO_2_ Concentration	Mass Transfer Performance	Main Conclusions
nanobubble membrane	Deionized water (DW)	NTA	≤5 days (The loss was over 60% after three days)	4~4.5	75–250 nm	−3.05 mV~+2.68 mV	——	CO_2_ MNBs are 11 times that of macroscopic large bubbles	CO_2_ MNBs optimize mass transfer and buffering simultaneously through their ultra-high specific surface area and interfacial reactivity [30].
	DW, Tap water (TW)	Zetasizer Nano ZS, ASTM D513-92 (1996) standard measurement	——	3.78 (TW); 2.13 (DW)	18.17 nm~299.5 nm (TW); 1.63 nm~216.1 nm (DW)	−5.91 mV (TW); −3.23 mV (DW)	It rose from 2 ppm to 24 ppm	——	CO_2_ MNBs and biochar work in synergy to achieve the triple goals of CO_2_ resource utilization, soil quality improvement, efficiency enhancement, and crop yield increase [32].
	DW	DLS, Zetasizer, CO_2_ Sensor	≥7 days	<4.0	200–500 nm	−8~−19 mV	2000 ppm	——	The high solubility and acidic environment of CO_2_ MNBs can provide a basis for food processing applications [39].
periodic transformer device	DW	DLS	<48 h	——	41 nm and 338 nm	——	——	——	CO_2_ MNBs take advantage of their high solubility to achieve a small size advantage, but their chemical activity limits their long-term stability [9].
high-pressure fluid dynamics	DW	Zetasizer, High-temperature NMR imaging analyzer	——	——	255~712 nm	−3.68 mV	——	A longer relaxation time table of CO_2_ MNBs than DW indicates an increase in the fluidity of water molecules	The addition of CO_2_ MNBs can significantly increase the methane yield of anaerobic digestion of corn stalks (up to 17%), and the mechanisms include enhancing microbial enzyme activity, improving pH buffering capacity, and promoting substrate degradation [36].
the hydrodynamic cavitation method	DW, 1 mM salt water (NaCl, CaCl_2_, AlCl_3_)	DLS, the phase analysis light scattering method	≤5 days (DW); ≥14 days (1 mM salt water)	4~4.5	160 nm (DW); 200~300 nm (1 mM salt water)	+9 mV (DW); +10 mV (1 mM salt water)	——	——	The stability of CO_2_ MNBs in salt water is enhanced due to ion inhibition dissolution [31].
mechanical high-speed cavitation equipment (self-made equipment)	Aqueous solution of ethanol (10%, 30%, 50% ethanol)	DLS, NTA, the phase analysis light scattering method	20 days	4~5	500 nm (10%); 3500 nm (50%);	−5 mV~0 mV;	——	——	The stability of CO_2_ MNBs is highly dependent on pH and ethanol concentration. When acidity collapses, hydroxyl radicals are produced, but a mixture of H_2_ can inhibit free radicals [33].
mechanical shear combined with pressure drop nucleation method	DW	Zeta Potential Analyzer	——	4.0	——	−20~−27 mV	——	——	The type of gas affects the stability of MNBs by altering the zeta potential. Among them, O_2_ MNBs and N_2_ MNBs are the most stable due to their high negative charges, while the stability of CO_2_ MNBs is weakened by an acidic environment [34].

## 3. Applications of CO_2_ MNBs in Different Fields

While the formation mechanisms underlying CO_2_ MNBs’ unique properties remain under active investigation, CO_2_ MNBs have demonstrated extraordinary potential across diverse aspects. Notable applications span agriculture, food processing, enhanced oil recovery, green construction, and mineral flotation, each demonstrating outstanding improvements in process sustainability and economic viability. This section systematically discusses these frontier applications and analyzes how they bring innovation and improvement in their respective fields.

### 3.1. Agriculture

As the primary substrate for plant photosynthesis, CO_2_ serves as the critical mediator for converting solar energy into biomass within agroecosystems, with its bioavailability directly governing crop growth efficiency and yield potential. Building upon the distinct physicochemical advantages of MNB technology, researchers began to explore innovative applications of CO_2_ MNBs in agriculture.

#### 3.1.1. Enhanced Gas Transfer and Carbon Source Delivery

Due to their huge specific surface area and very low buoyancy, CO_2_ MNBs can remain stable in water for extended periods. This stability significantly enhances both the saturation concentration and retention time of dissolved CO_2_. Consequently, the system continuously supplies high concentrations of CO_2_ to plant roots. This not only directly increases the substrate availability for photosynthesis but also promotes root respiration and nutrient uptake.

In 2018, a study employed a nanoporous membrane to generate air, O_2_, N_2_, and CO_2_ MNBs solutions for comparative analysis of their effects on lettuce, carrot, fava bean, and tomato. Germination assays (6–10 days) revealed that all gas-infused MNB treatments enhanced germination rates relative to tap water controls, with N_2_ MNBs demonstrating maximum effect (100% germination after 6-day immersion), followed by CO_2_ MNBs (85%), both exceeding the tap water control (80%). Subsequent plant growth experiments showed that N_2_ MNBs significantly promoted the growth of all tested plants (fava bean, carrot and tomato) after continuous irrigation of MNB water every three days, while CO_2_ MNBs specifically enhanced the growth performance of fava bean and tomato, showing that the morphological indexes such as leaf number, stem length, stem diameter, and leaf length and width increased synchronously [22].

**Figure 3:**
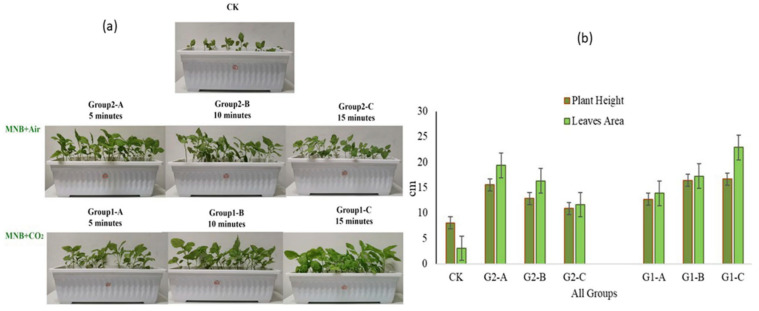
(**a**) The plants treated with tap water served as the control group(CK) and MNBs-treated plants; (**b**) Effects of air MNBs and CO2 MNBs on the plant height and leaf area of amaranth; Groups 2-A, B, and C are, respectively, air MNBs circulating in the MNB generator for 5 min, 10 min, and 15 min; Groups 1-A, B, and C are, respectively, CO_2_ MNBs circulating in the MNB generator for 5 min, 10 min, and 15 min [46].

**Figure 4:**
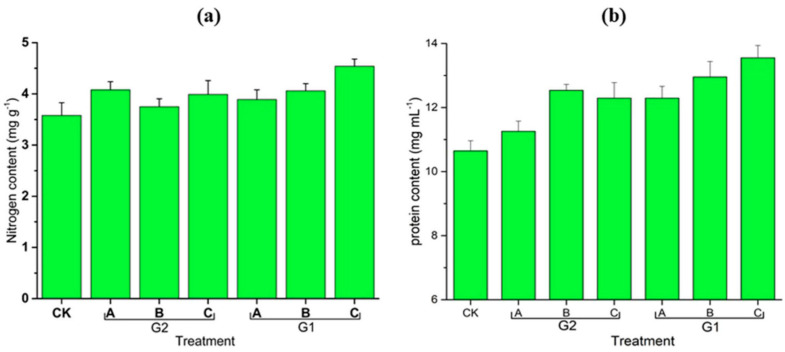
The effects of air MNBs and CO_2_ MNBs on the nitrogen content (**a**) and protein (**b**) of amaranth (CK was the control group of tap water; G2-A, B, and C were, respectively, air MNBs that circulated in the MNB generator for 5 min, 10 min, and 15 min; G1-A, B, and C were, respectively, CO_2_ MNBs that circulated in the MNB generator for 5 min, 10 min, and 15 min [46].

With the deepening of research, CO_2_ MNBs’ ability to enhance both germination rate and nutritional quality in amaranth becomes increasingly evident [46]. As shown in Figure 3 and Figure 4, treated plants exhibited optimized phenotypic indices (stem height, leaf area, root length) and increased accumulation of secondary metabolites (amino acids, vitamin C, soluble sugars, protein), showing positive correlations with MNB generator cycling time. The latest field trials have confirmed that the application of CO_2_ MNBs technology in the cultivation of tomatoes and kidney beans can increase the seed germination rate by 20% [32].

#### 3.1.2. Surface Negative Charge

The negative charge present on the surface of CO_2_ MNBs constitutes the core mechanism enabling their multifunctionality. Firstly, through electrostatic interactions, these surface charges effectively adsorb cationic nutrients (e.g., K^+^, Mg^2+^, NH_4_^+^) from the soil solution, significantly enhancing nutrient bioavailability [47]. The adsorbed cations migrate with the CO_2_ MNBs and accumulate within the plant rhizosphere, effectively mitigating the nutrient leaching loss common in traditional irrigation practices [32]. Secondly, the surface negative charges may also indirectly influence rhizosphere microbial community structure and function by affecting microbial cell membrane permeability. This effect was demonstrated in anaerobic fermentation, where CO_2_ MNBs increased microbial electron transport system activity by 23% and concurrently enhanced key enzyme activity. This ultimately improved corn stalk methane production efficiency by 4% to 17% while reducing secondary pollution risk [36]. In amaranth cultivation experiments, CO_2_ MNBs enhanced root cell membrane permeability, thereby improving the root’s capacity for water and nutrient uptake. This consequently promoted increases in stem height and leaf area expansion in amaranth [46].

#### 3.1.3. Soil Acidification Mitigation and Nutrient Mobilization

CO_2_ MNBs significantly extend the dissolution time of CO_2_ in water, enabling the stable formation of carbonic acid. This process leads to a substantial decrease in water pH. Within the resulting acidic aqueous environment, insoluble soil nutrients (such as phosphate) and trace elements (e.g., Zn, Mn, Mg) dissolve and become efficiently activated [32,46]. Concurrently, the HCO_3_^−^/CO_3_^2−^ buffer system, formed synchronously upon CO_2_ MNB dissolution, plays a crucial role. This buffer effectively mitigates potential sharp pH drops during the acidification stage [36]. Consequently, it promotes nutrient dissolution and activation while maintaining a relatively stable microenvironment more favorable for nutrient availability.

### 3.2. Food Processing

Within the food industry, the functional applications of food-grade gases such as CO_2_, N_2_, O_2_, and their mixtures have expanded from the traditional processing, packaging, and storage to the nanoscale control field. Particularly, CO_2_ remains pivotal for texture modification and shelf-life extension in carbonated beverages and dairy products, attributed to its high aqueous solubility (1.45 g/L at 25 °C), chemical inertness, and purification feasibility. Leveraging their unique physicochemical characteristics, CO_2_ MNBs are revolutionizing food processing through breakthrough applications in component separation, rheology control, crystallization optimization, and interfacial engineering.

#### 3.2.1. Efficient Nucleation Medium

**Figure 5:**
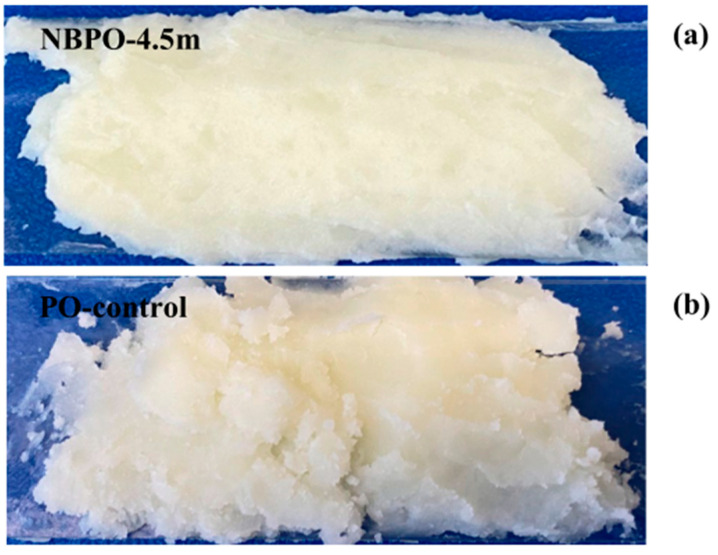
Visual observation of palm oil with (**a**) and without (**b**) CO_2_ MNBs [55].

Truong’s research group has pioneered systematic investigations into CO_2_-mediated targeted regulation of fat and sugar crystallization for food applications since 2017. Initial studies revealed CO_2_’s exceptional solubility in anhydrous milk fat (AMF), demonstrating its capacity to fundamentally alter crystallization kinetics: by promoting nucleation and accelerating α-type fat crystals formation, CO_2_ effectively modulated AMF’s macroscopic hardness through concurrent crystal size reduction and population enhancement [48,49]. These findings established critical physicochemical foundations for dairy product microstructure and rheological properties. Building upon this foundation, the team utilized the mechanical method (mechanical stirring or ultrasonication) to generate CO_2_ MNBs. In supersaturated lactose solutions, CO_2_ MNBs functioned as highly efficient nucleation sites, inducing morphological transition of α-lactose monohydrate crystals from tomahawk configurations to regular triangular or trapezoidal geometries. At the same time, the crystal yield is increased, and the crystal size is reduced without affecting the crystal density field [50]. This conclusion is consistent with the findings in similar systems [51]. In the follow-up work, the team verified the universality of CO_2_ MNBs technology in butter processing and ultrasonic-assisted AMF crystallization [52,53,54]. The latest research in 2024 shows that CO_2_ MNBs generated in palm oil by nanoporous membrane method can significantly optimize its crystallization characteristics: at 23 °C and 4 °C, CO_2_ MNBs all initiate crystallization in advance, forming finer and more uniform crystals, while lowering the melting end temperature and increasing the melting enthalpy, and finally obtaining the crystallized product with enhanced surface gloss and soft texture as shown in Figure 5, which is of great value for the development of grease products such as butter and shortening [55]. The team further found that the nucleation effect of CO_2_ MNBs can be extended to the freezing field: in sugar solution containing solute, CO_2_ MNBs induced by ultrasound can effectively increase the nucleation temperature of ice crystals and shorten the freezing time by providing a large number of nucleation sites, which is confirmed in the gelatin system [56,57]. Cross-system verification shows that CO_2_ MNBs can accelerate the freezing setting of ice cream, concentrated apple juice, and milk, and improve their texture softness and anti-melting, which highlights the multifunctional regulation potential of CO_2_ MNBs in food processing and freezing engineering [58,59]. A series of research by the Truong team revealed that CO_2_ MNBs had precisely controlled crystal morphology, size, and phase transition through a physical nucleation mechanism, which provided important theoretical support for green processing technology innovation.

#### 3.2.2. Synergistic Effect of High Stability and Surface Negative Charge

**Figure 6:**
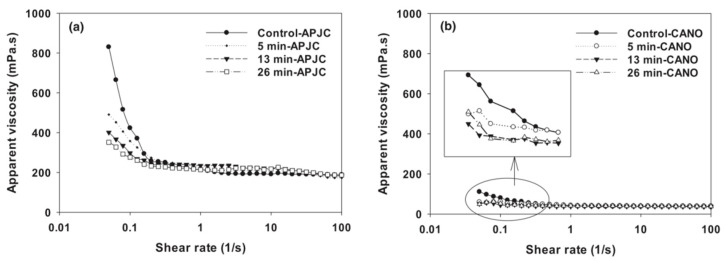
Apparent viscosity of concentrated apple juice APJC (**a**) and rapeseed oil (CANO) (**b**) after treatment with CO_2_ MNBs and their controls. Time is the cycle time of the MNBs’ generator field [61].

In the field of regulating the rheological properties of food, it was discovered that introducing air, N_2_, O_2_, CO_2_, and their mixed gases into the dairy products system can significantly reduce the viscosity of liquid and maintain the stability effect for three days [60]. It is pointed out that this effect is not only due to the high stability of CO_2_ MNBs, but also related to the negative charge characteristics carried on their surfaces: CO_2_ MNBs, casein, and other particles with similar charges in the solution produce polarity synergy and form a buffer layer between nanoparticles through charge interaction, which promotes particle separation and inhibits aggregation behavior, thus reducing the viscosity of the system. As shown in Figure 6, generated CO_2_ MNBs in apple juice concentrate and canola oil via nanoporous membranes achieved 18% and 10% viscosity reduction, respectively, and persisted for seven days without altering pH, density, or soluble solids [61]. These findings collectively revealed the dual advantages of CO_2_ MNBs: on the one hand, it optimizes the fluidity of food processing fluid through the physical mechanism to improve production efficiency; on the other hand, it avoids the use of traditional chemical additives, which provides an innovative path for developing cleanable food and realizing environment-friendly production.

Truong’s team recently pioneered a breakthrough using nanoporous membranes to generate CO_2_ MNBs in apple juice systems, demonstrating that CO_2_ MNBs can significantly reduce the surface tension of liquid and have no significant effect on the density of the matrix field [62]. This discovery provides an experimental basis for physical modification technology to replace chemical surfactants. Although the current research has been confirmed in the apple juice system, the universality of this technology to the surface tension of other food liquids still needs to be systematically verified. By using the interfacial activity characteristics of CO_2_ MNBs, the food processing performance can be optimized without introducing additives, which is of dual significance to food development and sustainable production mode construction in clean label.

#### 3.2.3. Enhanced Interfacial Enrichment via Ultra-Low Buoyancy

In the field of food component separation technology, it is found that the hydrophobic region of hydrophobic can form a complex with CO_2_ molecules through intermolecular forces, and self-assembly generates MNBs (110–120 nm). Taking advantage of this feature, the research team used CO_2_ foam fractionation to extract protein after its fermentation, which successfully increased the concentration of hydrophobic compounds by four times. In this process, although nano-spray drying led to the partial decrease in protein activity, the inactivation phenomenon was effectively suppressed by pre-adding surfactants. Because of the low density of CO_2_ MNBs, the enrichment of CO_2_ MNBs at the interface of the two phases increased the purity of hydrophobic by 2.8 times. This method not only simplifies the chromatographic purification steps, reduces the cost, and maintains the activity of hydrophobic, but also shows the technical breakthrough of CO_2_ MNBs in the field of biomolecule separation [63].

### 3.3. CO_2_ EOR and CGS

With the continuous growth of global energy demand and the gradual depletion of conventional oil and gas resources, reservoir development faces serious challenges. Deep saline formations and oil reservoirs—considered ideal sites for geological CO_2_ storage—exhibit declining recovery rates and limited remaining recoverable reserves due to their development stage having entered the middle and late stages. Conventional CO_2_-EOR remains constrained by technical bottlenecks such as gas channeling, gravity override, and insufficient gas–reservoir contact time, further exacerbating energy supply limitations. Meanwhile, increasingly stringent environmental regulations have highlighted the carbon emissions of oil and gas development. In this part, CO_2_ MNBs effectively suppress viscous fingering and gravitational override phenomena, enhance sweep efficiency and volumetric displacement of CO_2_, and simultaneously improve CO_2_-EOR and CGS efficacy. This technology has recently gained traction as an innovative direction in oil and gas extraction.

#### 3.3.1. Micro-Nano Size and Efficient Mass Transfer

The development history of CO_2_ MNBs in CO_2_-EOR and CGS has shown significant phased breakthroughs. Early basic research verified the permeation enhancement effect of CO_2_ MNBs in microporous media [64]. Their nanoscale size and reduced capillary force can effectively expand the contact area at the CO_2_-crude oil or water interface [65]. Subsequent simulations and experiments confirmed that supercritical CO_2_ MNBs can achieve a uniform distribution of high saturation in saline porous media, thereby improving sweep efficiency and mitigating gravitational override [66]. The pore-scale observation of Berea sandstone through X-ray computed tomography revealed the mechanism by which CO_2_ MNBs activate the low-permeability area by broadening the flow channel network [67]. Systematic studies have further clarified that CO_2_ MNBs offer dual advantages in enhancing crude CO_2_-EOR and improving CGS safety by strengthening mass transfer efficiency and dissolution kinetics [68,69,70,71,72]. In recent years, it was found that CO_2_ MNBs can not only improve the CO_2_-EOR efficiency by contacting the residual oil on the pore wall, but also dynamically regulate the seepage path by using the “Jamin effect”, with the help of a visual microfluidic platform [73].

#### 3.3.2. High Stability

The latest experiments show that CO_2_ MNBs, by modifying nano-SiO_2_ particles, maintained the size stability by electrostatic adsorption and hydrogen bonding, and inhibited CO_2_ overflow. The constructed system has the advantages of temperature resistance, oil resistance, dimensional stability, and anti-wetting. The oil displacement experiment shows that the oil recovery ratio is increased by 17.64% compared with conventional CO_2_-EOR, and the risk of CO_2_ channeling is significantly reduced [74].

**Figure 7:**
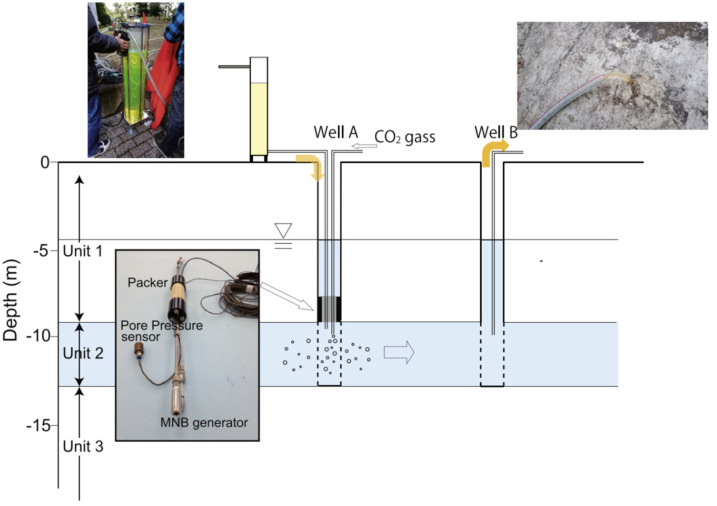
Schematic diagram of a small-scale in situ injection test. The CO_2_ gas is conveyed to the MNB generator through the packer and forms bubbles in Well A [35].

The high stability and low buoyancy of CO_2_ MNBs enable long-term discrete-phase storage in formation water, substantially lowering CGS leakage risks [70]. Field studies of Tokyo’s Setagaya-ku shallow aquifers revealed that injected CO_2_ MNBs persist for one day while gradually displacing groundwater(Figure 7) [35]. Notably, CO_2_ MNBs generation requires no chemical additives, aligning with green development principles. Although the understanding of CO_2_ MNBs’ stability and phase transitions under high-temperature and high-pressure conditions requires further investigation, their demonstrated recovery enhancement and storage safety have been extensively validated.

#### 3.3.3. Efficient Nucleation Medium

As a clean energy resource possessing reserves that far exceed traditional fossil fuels, natural gas hydrate extraction faces multifaceted challenges in exploration, technological implementation, and environmental risk management. Research reveals that CO_2_ MNBs accelerate hydrate formation by increasing nucleation frequency through shortened induction times [75].

#### 3.3.4. Surface Negative Charge

Furthermore, CO_2_ MNBs demonstrate unique potential in microbial enhanced oil recovery: their surface charge characteristics enhance cation mass transfer efficiency in anaerobic environments. This mechanism enhances both the microbial activity and metabolite production [76,77].

Comprehensively, CO_2_ MNBs represent a breakthrough, addressing traditional technological limitations through multidimensional innovation. They demonstrate distinctive advantages in enhancing CO_2_-EOR, strengthening CGS, facilitating hydrate development, and advancing microbial processes. With ongoing refinements in MNB fundamental theories and engineering applications, this technology promises to catalyze sustainable development in petroleum extraction.

### 3.4. Construction and Mining

Extending the concrete structure has emerged as a pivotal strategy for reducing environmental impacts and optimizing resources in construction. While CO_2_-induced calcium ion carbonation generates pore-filling calcium carbonate, simultaneously densifying concrete surfaces and enabling crack self-healing, active regulation of atmospheric carbonation processes remains technically challenging.

#### 3.4.1. Enhanced Gas Transfer and Carbon Source Delivery

**Figure 8:**
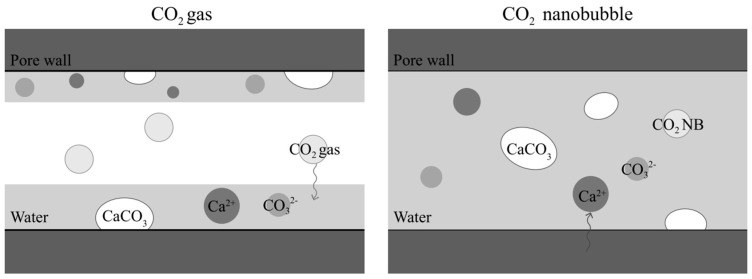
Schematic representation of carbonization of CO_2_ gas (**left**) and CO_2_ MNBs (**right**) [79].

Recent studies have shown that CO_2_ MNBs can significantly enhance the carbonization efficiency and optimize the material repair performance. Through experimental verification, concurrent calcium carbonate formation on both crack surfaces and interior regions significantly boosts self-healing capacity [78]. Further research has found that CO_2_ MNBs rapidly fill pores and accelerate precipitation reactions, achieving mass transfer efficiency far surpassing conventional CO_2_ gas (Figure 8) [79]. They innovatively combined calcium nitrite with CO_2_ MNBs water, revealing that structural densification via increased calcium carbonate production effectively reduces carbonation depth and porosity while enhancing mechanical properties [80].

In construction waste treatment, CO_2_ MNBs technology exhibits distinctive environmental benefits by utilizing its acidic properties to neutralize alkaline concrete wastewater, achieving a 1–2% increase in compressive strength of recycled cement paste [81]. Experimental studies show that CO_2_ MNBs enhance calcium carbonate deposition in recycled cement paste by 110%, dramatically reduce reaction duration, and boost CO_2_ utilization efficiency from 31.9% to 82.2%, with concurrent increases in specific surface area of porous calcium carbonate particles [82].

For mine filling materials, cementitious composites incorporating CO_2_ MNBs were developed to optimize pore structure. These materials exhibit improved compressive strength and hydration stability after 28-day curing, offering a novel low-carbon solution for sustainable mining [83,84].

#### 3.4.2. Surface Negative Charge

CO_2_ MNBs exhibit multi-scale collaborative regulation capabilities in the mineral flotation system. For pyrite flotation, CO_2_ suppresses surface oxidation to maintain hydrophobicity while enhancing flotation recovery [85]. Subsequent work revealed rapid CO_2_ MNBs formation on pyrite surfaces in CO_2_-saturated solutions [86]. Atomic force microscopy and molecular dynamics simulations identified unique CO_2_ MNBs adhesion and diffusion mechanisms, facilitating millimeter-scale N_2_ bubble attachment through multimolecular structuring and synergistically reducing surface oxidation. Lian et al. pioneered a hybrid approach combining CO_2_ MNBs with microemulsion trapping: Firstly, microemulsion is used to improve the surface hydrophobicity of low-rank coal, and then, CO_2_ MNBs are induced into the flotation system, which can be selectively adsorbed on the surface of low-rank coal through its surface charge, thus weakening the electrostatic repulsion between coal particles, promoting the aggregation of coal particles and improving the collision probability between bubbles and particles, and finally, realizing the systematic optimization of flotation performance [87].

Comprehensive research shows that CO_2_ MNBs water forms a technical closed loop in enhancing the performance of building materials, reducing cement consumption, carbon sequestration, and emission reduction, and recycling waste through the synergistic effect of efficient mass transfer and chemical reaction, which opens up an innovative path for realizing the sustainable development of the construction industry and mining industry.

### 3.5. Anaerobic Digestion

With the increasing global focus on renewable energy and environmental protection, anaerobic digestion has garnered significant attention for its dual advantages in simultaneous waste treatment and energy recovery. This technology utilizes microorganisms to decompose organic matter in an anoxic environment, producing renewable biogas (primarily methane) that can be used for power or heat generation. However, substrate degradation, microbial activity, and reaction conditions often constrain its efficiency, making improving digestion efficiency and gas production a critical research priority. Recent advancements in CO_2_ MNBs technology have introduced an innovative approach to address these limitations.

#### 3.5.1. Enhanced Gas Transfer and Carbon Source Delivery

Early basic research first confirmed that injecting CO_2_ MNBs into waste-activated sludge significantly enhanced biogas and methane production. The mechanism of action stems from the enhanced nutrient transfer efficiency at the bubble interface, thereby accelerating substrate decomposition [88]. This discovery lays the foundation for subsequent technological expansion: In the anaerobic digestion system of pig manure, CO_2_ MNBs not only increase the gas production, but also accelerate the hydrolysis and acidification process and improve the biological activity of the system by enhancing the respiratory activity of microorganisms [89].

#### 3.5.2. Buffering pH Fluctuation and Stabilizing Anaerobic Digestion

Further mechanism research revealed the steady-state regulatory function of CO_2_ MNBs: by dynamically buffering pH fluctuations and promoting the conversion of volatile fatty acids, thereby mitigating acid accumulation and methane inhibition. Molecular-scale analysis indicated that CO_2_ MNBs enhanced hydrolysis enzyme activity while optimizing water molecule mobility and nutrient transfer efficiency [90]. Expanding on these findings, verifying that CO_2_ MNBs reduced cellulose crystallinity through intensified microbial metabolic activity on high-loading cellulosic systems, concurrently improving hydrolysis-acidification efficiency and methane yield on high-loading cellulosic systems [91].

In summary, CO_2_ MNBs offer a critical optimization pathway for anaerobic digestion technology through three key mechanisms: pH regulation, enhancement of microbial metabolic environments, and improved substrate decomposition efficiency. The distinctive physicochemical characteristics of CO_2_ MNBs not only facilitate the efficient conversion of organic matter into biogas with reduced treatment costs and environmental risks but also establish a novel strategy for achieving resource recovery from organic waste. This dual functionality positions the technology as a promising solution with broad application prospects in both environmental protection and sustainable energy sectors.

### 3.6. Microalgae Cultivation

Microalgae utilize atmospheric CO_2_ through photosynthesis while assimilating nutrients such as carbon, nitrogen, and phosphorus from the water column. This dual functionality reduces atmospheric greenhouse gas concentrations and mitigates aquatic eutrophication. As a versatile feedstock for high-value-added products, including biofuels, vitamins, and biochar, microalgae demonstrate significant economic and environmental potential in climate change mitigation, driving growing global demand. However, ambient CO_2_ limitations and pH elevation induced by photosynthesis constrain natural carbon sequestration rates in microalgae. Elevated pH shifts inorganic carbon speciation toward HCO_3_^−^ and CO_3_^2−^, thereby reducing dissolved CO_2_ availability and ultimately impairing carbon fixation efficiency.

#### Enhanced Gas Transfer and Carbon Source Delivery

To address carbon utilization inefficiencies, researchers have investigated CO_2_ supplementation techniques to enhance the algal CO_2_ utilization ratio while lowering water pH [92,93,94]. At present, the most common supplement technology is the continuous bubbling method. Continuously bubbling air containing 10% CO_2_ into the algae membrane bioreactor improves the productivity and denitrification capacity of microalgae by 40% and reduces membrane pollutants in the algae membrane bioreactor [95]. However, the mass transfer efficiency of millimeter-to-centimeter bubbles generated by the traditional bubbling method is low, and the utilization rate of CO_2_ is limited. This limitation has spurred innovation in aerator design. The latest research boosted biomass productivity by 33.33% through bubble size reduction (27.97% smaller diameter, 46.88% lower rise velocity) by optimizing the aeration device [96]. Previous studies on optimizing aeration devices have jointly confirmed the promoting effect of smaller bubbles on growth, although these studies have all focused on millimeter-scale systems [97,98,99,100,101,102].

Early studies used MBs (air containing 20% CO_2_) of 300 μm and 500 μm for the first time to cultivate Dunaliella salina, and its mass transfer efficiency, dissolved oxygen removal ability, growth rate, and biomass density were significantly improved [103]. Later, a study uses CO_2_ MBs to obtain a similar conclusion [104]. However, research points out that the growth efficiency of microalgae is related to the radiation characteristics of bubbles. Experiments show that when the bubble volume fraction is 0.003 and the radius is 3.5 mm, microalgae growth and CO_2_ fixation are the best [105]. The latest technological breakthrough focuses on the application of MNBs. CO_2_ MNBs generated by ceramic nozzles under acidic conditions, with their high density and stability, retain more non-ionic CO_2_ in the culture medium, which promotes the cell yield and vitamin content of green algae to increase by 1.78 times and 1.5 times, respectively [106].

It is worth noting that all the CO_2_ MNBs studied above are MNBs produced by the gas mixed with CO_2_ and air or nitrogen. In the future, we can continue to explore the influence of mixing different concentrations of CO_2_ on algae growth in the field of microalgae culture. CO_2_ MNBs can not only enhance gas–liquid mass transfer but also increase the concentration of dissolved carbon in suspension. It is believed that CO_2_ MNBs can break through the traditional carbon source supply limitation in the field of microalgae culture, significantly improve the photosynthetic efficiency and biomass accumulation rate, and at the same time reduce the energy consumption cost by optimizing the carbon capture process, thus providing efficient and sustainable technical support for the large-scale production of high value-added microalgae products and the realization of carbon neutrality.

### 3.7. Industrial Crystallization

In addition to the application of optimizing crystallization in the food field, CO_2_ MNBs, as a new green additive that does not introduce external impurities and does not participate in the reaction, have also received extensive attention in the industrial field in recent years.

#### 3.7.1. Efficient Nucleation Medium

Early basic research induced nucleation of CO_2_ bubbles through rapid pressure reduction at the gas–liquid interface, found that it can increase nucleation active sites, thereby accelerating ultrafine particle generation [107]. This principle was subsequently extended to polycrystalline systems: in complex systems such as curcumin, poultry pulp, piroxone, and cholesterol, CO_2_ MNBs demonstrated nucleation-promoting effects, verifying its technical applicability across substance categories [108,109,110,111]. An early method was developed to prepare Li_2_CO_3_ nanoparticles by microwave irradiation of CO_2_ MNBs’ solution. It was found that the crystallization rate of nanoparticles could be significantly improved by adjusting the bubble size and heating rate, in which the smaller the bubble size, the higher the heating rate and the stronger the crystallization rate, indicating that CO_2_ MNBs as a green additive can optimize the mass transfer process of cooling crystallization system, thus improving the nucleation rate and crystal growth [112]. Huang et al. identified a dual role of CO_2_ MNBs in high-temperature supersaturated solutions: while surface-adherent bubbles may physically disrupt crystal formation, their dominant effect enhances solution thermodynamics and kinetics, ultimately promoting crystallization efficiency [113,114].

#### 3.7.2. Surface Negative Charge

In addition, the CO_2_ MNBs proposed by Kimura et al. can adsorb cations with negative charges on their surfaces, forming local supersaturated regions, thus inducing the formation of finer crystal particles [115]. The common surface of these studies is that CO_2_ MNBs can be used as efficient and environmentally friendly crystallization control means under different mechanisms.

## 4. Summary and Outlook

With unique physicochemical properties, CO_2_ MNBs have emerged as a transformative technology with cross-industrial applications. This comprehensive review systematically analyzes their innovative implementations in agriculture, food processing, oil/gas extraction, construction, mining, anaerobic digestion, microalgae cultivation, and industrial processes. CO_2_ MNBs reveal their excellent advantages of upgrading the traditional technology through high mass transfer efficiency, high stability, and surface negative charge. Among these properties, stability and surface negative charge play critical roles. Long-term stability is essential for ensuring CO_2_ MNBs maintain their function over extended periods, enabling them to sustain both a high-concentration CO_2_ microenvironment and high mass transfer efficiency. The surface negative charge drives specific interactions (such as electrostatic attraction or repulsion) between CO_2_ MNBs and other environmental components, thereby influencing related reaction processes and underlying mechanisms. It is particularly noteworthy that CO_2_ MNBs technology realizes the efficient utilization of gas resources through physical means, which provides a green technical path for the realization of carbon neutrality.

Although great progress has been made in the application research of CO_2_ MNBs, many characteristics and preparation aspects of CO_2_ MNBs are still relatively lacking. The following aspects can be further studied in the future: (1) To deepen the theoretical research, establishing the kinetic model of CO_2_ MNBs and the quantitative description of the interfacial reaction mechanism; (2) To develop a large-scale generator with high efficiency and low energy consumption to break through the technical bottleneck of engineering application; (3) To expand multi-technology collaborative application scenarios and expand the application scope of CO_2_ MNBs and explore the coupling innovation of CO_2_ MNBs with electrochemical catalysis, membrane separation, and other technologies; (4) To establish an industry standard system and formulate technical specifications and safety assessment standards for different application scenarios. With the collaborative breakthrough of basic research and engineering technology, CO_2_ MNBs technology is expected to develop into an important technical platform to support green manufacturing and sustainable development.
